# 1,3-Diethyl-2-sulfanyl­idene-5-(2,4,5-trimeth­oxy­benzyl­idene)-1,3-diazinane-4,6-dione

**DOI:** 10.1107/S1600536812049707

**Published:** 2012-12-08

**Authors:** Abdullah M. Asiri, Muhammad Nadeem Arshad, Muhammad Zia-ur-Rehman, Tariq R. Sobahi

**Affiliations:** a Chemistry Department, Faculty of Science, King Abdulaziz University, PO Box 80203, Jeddah 21589, Saudi Arabia; b Center of Excellence for Advanced Materials Research (CEAMR), Faculty of Science, King Abdulaziz University, PO Box 80203, Jeddah 21589, Saudi Arabia; c Applied Chemistry Research Centre, PCSIR Laboratories Complex, Ferozpure Road, Lahore 54600, Pakistan

## Abstract

The title compound, C_18_H_22_N_2_O_5_S, is largely planar, with an r.m.s. deviation of 0.0546 (1) Å of atoms from the mean plane through all non-H atoms except for the methyl groups. The benzene and pyrimidine­dione rings are inclined to one another at a dihedral angle of 1.41 (7)°. In the crystal, weak C—H⋯O inter­actions connect the mol­ecules into chains propagating along the *b-*axis direction.

## Related literature

For the synthesis of the title compound, see: Asiri *et al.* (2004 ▶). For a related structure, see: Asiri *et al.* (2009 ▶).
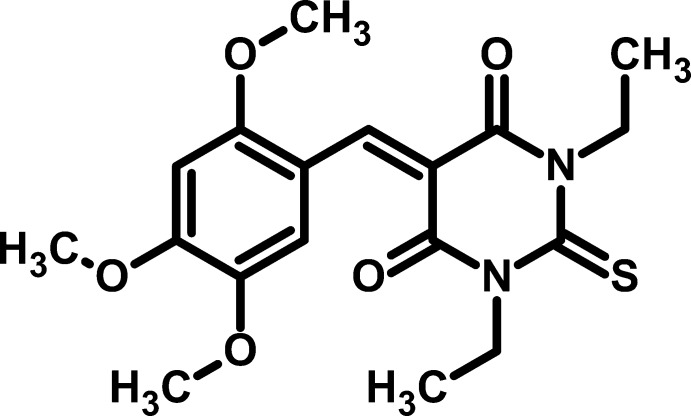


## Experimental

### 

#### Crystal data

C_18_H_22_N_2_O_5_S
*M* *_r_* = 378.44
Monoclinic, 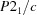

*a* = 7.9711 (1) Å
*b* = 17.4106 (3) Å
*c* = 13.5265 (2) Å
β = 99.237 (2)°
*V* = 1852.89 (5) Å^3^
*Z* = 4
Cu *K*α radiation
μ = 1.83 mm^−1^
*T* = 296 K
0.29 × 0.10 × 0.09 mm

#### Data collection

Agilent SuperNova (Dual, Cu at zero, Atlas, CCD) diffractometer
Absorption correction: multi-scan (*CrysAlis PRO*; Agilent, 2012 ▶) *T*_min_ = 0.875, *T*_max_ = 1.000
14850 measured reflections
3777 independent reflections
3083 reflections with *I* > 2σ(*I*)
*R*_int_ = 0.027

#### Refinement

*R*[*F* ^2^ > 2σ(*F* ^2^)] = 0.041
*wR*(*F* ^2^) = 0.121
*S* = 1.05
3777 reflections
240 parameters
H-atom parameters constrained
Δρ_max_ = 0.25 e Å^−3^
Δρ_min_ = −0.20 e Å^−3^

### 

Data collection: *CrysAlis PRO* (Agilent, 2012 ▶); cell refinement: *CrysAlis PRO*; data reduction: *CrysAlis PRO*; program(s) used to solve structure: *SHELXS97* (Sheldrick, 2008 ▶); program(s) used to refine structure: *SHELXL97* (Sheldrick, 2008 ▶); molecular graphics: *PLATON* (Spek, 2009 ▶); software used to prepare material for publication: *WinGX* (Farrugia, 2012 ▶) and *X-SEED* (Barbour, 2001 ▶).

## Supplementary Material

Crystal structure: contains datablock(s) I, global. DOI: 10.1107/S1600536812049707/sj5286sup1.cif

Structure factors: contains datablock(s) I. DOI: 10.1107/S1600536812049707/sj5286Isup2.hkl

Supplementary material file. DOI: 10.1107/S1600536812049707/sj5286Isup3.cml

Additional supplementary materials:  crystallographic information; 3D view; checkCIF report

## Figures and Tables

**Table 1:** Hydrogen-bond geometry (Å, °)

*D*—H⋯*A*	*D*—H	H⋯*A*	*D*⋯*A*	*D*—H⋯*A*
C13—H13*C*⋯O2^i^	0.96	2.58	3.455 (2)	152

Symmetry code: (i) 
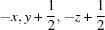
.

**Fig. 1.**
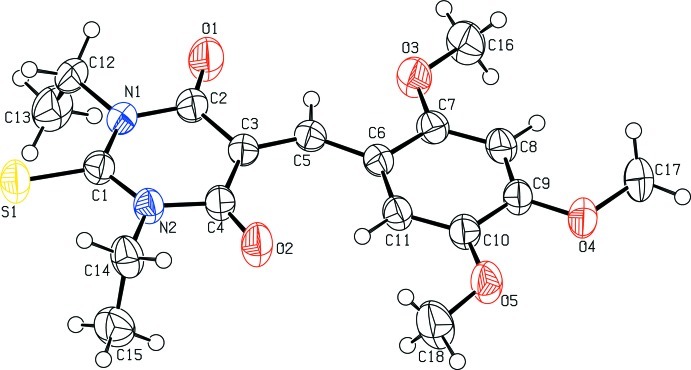
The structure of (I) with 50% probability ellipsoids.

**Fig. 2.**
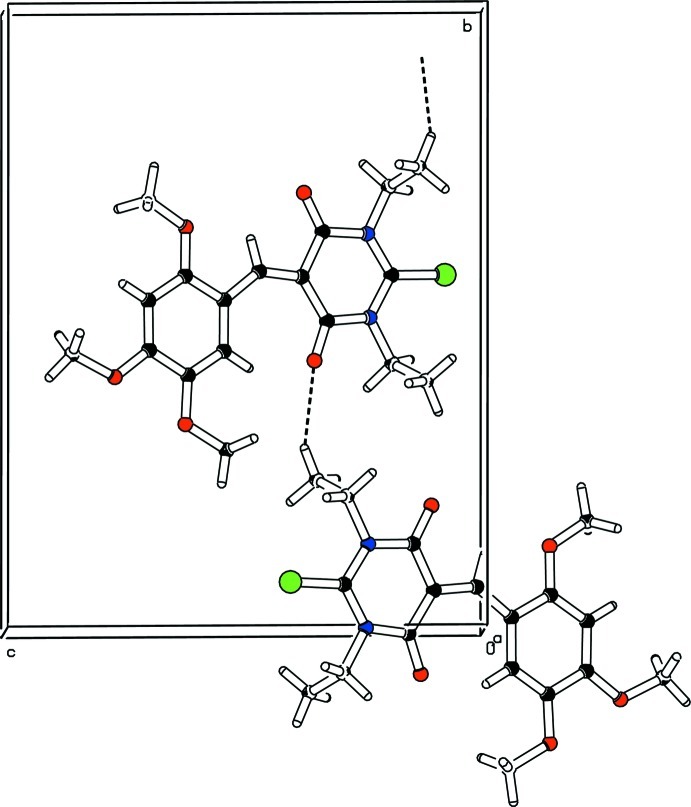
Unit cell diagram showing C—H···O hydrogen bonds, drawn as dashed lines.

## supplementary crystallographic information

### Comment

The title compound is related to the arylidene 5-[3-(2,5-dimethoxyphenyl)prop- 2-enylidene]-1,3-diethyl-2-thioxohexahydropyrimidine-4,6-dione (II) already reported by our group (Asiri *et al.*, 2009). The molecule is largely planar with a dihedral angle between the benzene and pyrimidine dione rings of 1.41 (7)°. The r. m. s. deviation of atoms from the best fit plane through the non-hydrogen atoms C1/N1/C2/C3/C4/N2/S1/O1/O2/C5/C6/C7/C8/C9/C10/C11/C12/ C14/O3/O4/O5) is 0.0546 (1) Å. Atoms S1 and O1 are displaced from this plane by -0.1187 (1) Å and 0.1131 (2) Å respectively. Non-classical C13–H13C···O2 hydrogen bonds connect the molecule into chains along the *b* axis (Table. 1, Fig. 2).

### Experimental

1,3-Diethyl-2-thiobarbituric acid (0.005 mol) and 2,4,5-trimethoxybenzaldehyde (0.005 mol) were heated in ethanol (15 ml) for 3 h; several drops of diethylamine were added. The progress of reaction was monitored by TLC. The mixture was cooled and the resulting solid was recrystallized from methanol (Asiri *et al.* 2004) by slow evaporation at room temperature.

### Refinement

All the H-atoms bound to C were positioned with idealized geometry with C—H = 0.93 Å for aromatic, C—H = 0.97 Å for methylene & C—H = 0.96 Å for methyl groups. H-atoms were refined as riding with *U*_iso_(H) = k*U*_eq_(C), where k = 1.5 for methyl H-atoms & k = 1.2 for other H-atoms .

### Crystal data

**Table:**

C_18_H_22_N_2_O_5_S	*F*(000) = 800
*M**_r_* = 378.44	*D*_x_ = 1.357 Mg m^−^^3^
Monoclinic, *P*2_1_/*c*	Cu *K*α radiation, λ = 1.54184 Å
Hall symbol: -P 2ybc	Cell parameters from 6212 reflections
*a* = 7.9711 (1) Å	θ = 3.3–75.4°
*b* = 17.4106 (3) Å	µ = 1.83 mm^−^^1^
*c* = 13.5265 (2) Å	*T* = 296 K
β = 99.237 (2)°	Needle like, red
*V* = 1852.89 (5) Å^3^	0.29 × 0.10 × 0.09 mm
*Z* = 4	

### Data collection

**Table:**

Agilent SuperNova (Dual, Cu at zero, Atlas, CCD) diffractometer	3777 independent reflections
Radiation source: SuperNova (Cu) X-ray Source	3083 reflections with *I* > 2σ(*I*)
Mirror monochromator	*R*_int_ = 0.027
ω scans	θ_max_ = 75.6°, θ_min_ = 4.2°
Absorption correction: multi-scan (*CrysAlis PRO*; Agilent, 2012)	*h* = −9→7
*T*_min_ = 0.875, *T*_max_ = 1.000	*k* = −21→21
14850 measured reflections	*l* = −16→16

### Refinement

**Table:**

Refinement on *F*^2^	Primary atom site location: structure-invariant direct methods
Least-squares matrix: full	Secondary atom site location: difference Fourier map
*R*[*F*^2^ > 2σ(*F*^2^)] = 0.041	Hydrogen site location: inferred from neighbouring sites
*wR*(*F*^2^) = 0.121	H-atom parameters constrained
*S* = 1.05	*w* = 1/[σ^2^(*F*_o_^2^) + (0.0597*P*)^2^ + 0.385*P*] where *P* = (*F*_o_^2^ + 2*F*_c_^2^)/3
3777 reflections	(Δ/σ)_max_ = 0.001
240 parameters	Δρ_max_ = 0.25 e Å^−^^3^
0 restraints	Δρ_min_ = −0.20 e Å^−^^3^

### Special details

**Table:**

Geometry. All esds (except the esd in the dihedral angle between two l.s. planes) are estimated using the full covariance matrix. The cell esds are taken into account individually in the estimation of esds in distances, angles and torsion angles; correlations between esds in cell parameters are only used when they are defined by crystal symmetry. An approximate (isotropic) treatment of cell esds is used for estimating esds involving l.s. planes.
Refinement. Refinement of F^2^ against ALL reflections. The weighted R-factor wR and goodness of fit S are based on F^2^, conventional R-factors R are based on F, with F set to zero for negative F^2^. The threshold expression of F^2^ > 2sigma(F^2^) is used only for calculating R-factors(gt) etc. and is not relevant to the choice of reflections for refinement. R-factors based on F^2^ are statistically about twice as large as those based on F, and R- factors based on ALL data will be even larger.

### Fractional atomic coordinates and isotropic or equivalent isotropic displacement parameters (Å^2^)

**Table:**

	*x*	*y*	*z*	*U*_iso_*/*U*_eq_	
S1	−0.22340 (6)	0.56530 (3)	0.08605 (3)	0.05970 (16)	
O1	0.0851 (2)	0.69234 (8)	0.38430 (11)	0.0875 (5)	
O2	0.10560 (19)	0.42242 (7)	0.36247 (9)	0.0651 (4)	
O3	0.40703 (17)	0.63202 (7)	0.63548 (9)	0.0591 (3)	
O4	0.58803 (17)	0.38883 (7)	0.78593 (9)	0.0595 (3)	
O5	0.42004 (19)	0.31584 (7)	0.63615 (9)	0.0646 (4)	
N1	−0.05338 (18)	0.62856 (8)	0.25049 (10)	0.0472 (3)	
N2	−0.05463 (17)	0.49412 (7)	0.24558 (10)	0.0435 (3)	
C1	−0.1055 (2)	0.56302 (9)	0.19924 (12)	0.0431 (4)	
C2	0.0503 (2)	0.63052 (10)	0.34507 (13)	0.0527 (4)	
C3	0.1117 (2)	0.55692 (9)	0.38958 (11)	0.0415 (3)	
C4	0.0594 (2)	0.48671 (9)	0.33594 (11)	0.0428 (3)	
C5	0.2186 (2)	0.56421 (8)	0.47980 (11)	0.0411 (3)	
H5	0.2339	0.6157	0.4973	0.049*	
C6	0.31256 (19)	0.51620 (9)	0.55427 (11)	0.0388 (3)	
C7	0.4108 (2)	0.55426 (9)	0.63690 (11)	0.0420 (3)	
C8	0.5034 (2)	0.51281 (10)	0.71486 (11)	0.0456 (4)	
H8	0.5662	0.5387	0.7686	0.055*	
C9	0.5032 (2)	0.43358 (10)	0.71329 (11)	0.0449 (4)	
C10	0.4101 (2)	0.39422 (9)	0.63098 (11)	0.0454 (4)	
C11	0.3173 (2)	0.43493 (9)	0.55483 (11)	0.0421 (3)	
H11	0.2552	0.4083	0.5015	0.050*	
C12	−0.1014 (3)	0.70471 (10)	0.20484 (14)	0.0615 (5)	
H12A	−0.1120	0.7414	0.2574	0.074*	
H12B	−0.2111	0.7007	0.1621	0.074*	
C13	0.0275 (3)	0.73339 (11)	0.14422 (17)	0.0764 (6)	
H13A	0.0335	0.6987	0.0897	0.115*	
H13B	0.1367	0.7364	0.1859	0.115*	
H13C	−0.0052	0.7834	0.1182	0.115*	
C14	−0.1166 (2)	0.42108 (10)	0.19685 (13)	0.0537 (4)	
H14A	−0.2290	0.4291	0.1586	0.064*	
H14B	−0.1263	0.3830	0.2480	0.064*	
C15	−0.0014 (3)	0.39104 (12)	0.12873 (15)	0.0670 (5)	
H15A	0.1113	0.3852	0.1656	0.101*	
H15B	0.0009	0.4265	0.0746	0.101*	
H15C	−0.0425	0.3422	0.1023	0.101*	
C16	0.5046 (3)	0.67409 (11)	0.71491 (15)	0.0659 (5)	
H16A	0.6227	0.6615	0.7185	0.099*	
H16B	0.4682	0.6610	0.7771	0.099*	
H16C	0.4887	0.7281	0.7027	0.099*	
C17	0.6693 (3)	0.42503 (13)	0.87605 (14)	0.0652 (5)	
H17A	0.5884	0.4565	0.9028	0.098*	
H17B	0.7613	0.4564	0.8616	0.098*	
H17C	0.7126	0.3865	0.9241	0.098*	
C18	0.3502 (4)	0.27476 (12)	0.54961 (16)	0.0889 (8)	
H18A	0.3996	0.2927	0.4936	0.133*	
H18B	0.2294	0.2826	0.5364	0.133*	
H18C	0.3739	0.2210	0.5598	0.133*	

### Atomic displacement parameters (Å^2^)

**Table:**

	*U*^11^	*U*^22^	*U*^33^	*U*^12^	*U*^13^	*U*^23^
S1	0.0627 (3)	0.0666 (3)	0.0436 (2)	0.0013 (2)	−0.01048 (19)	0.00529 (18)
O1	0.1387 (15)	0.0401 (7)	0.0671 (9)	0.0214 (8)	−0.0336 (9)	−0.0107 (6)
O2	0.0932 (10)	0.0372 (6)	0.0533 (7)	−0.0014 (6)	−0.0234 (7)	0.0007 (5)
O3	0.0709 (8)	0.0411 (6)	0.0572 (7)	−0.0051 (6)	−0.0145 (6)	−0.0083 (5)
O4	0.0676 (8)	0.0606 (7)	0.0434 (6)	0.0106 (6)	−0.0122 (5)	0.0023 (5)
O5	0.0981 (10)	0.0408 (6)	0.0467 (7)	0.0036 (6)	−0.0128 (6)	0.0039 (5)
N1	0.0545 (8)	0.0427 (7)	0.0412 (7)	0.0128 (6)	−0.0015 (6)	−0.0003 (5)
N2	0.0476 (7)	0.0419 (7)	0.0382 (6)	−0.0027 (6)	−0.0019 (5)	−0.0003 (5)
C1	0.0397 (8)	0.0492 (9)	0.0399 (8)	0.0026 (6)	0.0047 (6)	0.0014 (6)
C2	0.0670 (11)	0.0424 (9)	0.0441 (8)	0.0112 (8)	−0.0048 (8)	−0.0045 (7)
C3	0.0467 (8)	0.0389 (8)	0.0374 (7)	0.0037 (6)	0.0023 (6)	−0.0004 (6)
C4	0.0481 (9)	0.0403 (8)	0.0377 (7)	−0.0016 (6)	−0.0005 (6)	0.0020 (6)
C5	0.0459 (8)	0.0370 (7)	0.0394 (8)	0.0006 (6)	0.0037 (6)	−0.0036 (6)
C6	0.0391 (7)	0.0413 (8)	0.0352 (7)	−0.0016 (6)	0.0033 (6)	−0.0020 (6)
C7	0.0412 (8)	0.0420 (8)	0.0417 (8)	−0.0030 (6)	0.0033 (6)	−0.0041 (6)
C8	0.0417 (8)	0.0520 (9)	0.0401 (8)	−0.0026 (7)	−0.0027 (6)	−0.0073 (6)
C9	0.0427 (8)	0.0536 (9)	0.0363 (8)	0.0034 (7)	−0.0002 (6)	0.0023 (6)
C10	0.0538 (9)	0.0413 (8)	0.0390 (8)	0.0000 (7)	0.0009 (7)	0.0013 (6)
C11	0.0485 (9)	0.0412 (8)	0.0343 (7)	−0.0030 (6)	−0.0001 (6)	−0.0009 (6)
C12	0.0780 (13)	0.0455 (9)	0.0548 (10)	0.0238 (9)	−0.0076 (9)	−0.0010 (8)
C13	0.1027 (17)	0.0430 (10)	0.0783 (14)	0.0035 (11)	−0.0015 (12)	0.0120 (9)
C14	0.0613 (11)	0.0513 (9)	0.0444 (9)	−0.0132 (8)	−0.0039 (8)	0.0005 (7)
C15	0.0834 (15)	0.0549 (11)	0.0608 (11)	−0.0030 (10)	0.0053 (10)	−0.0092 (9)
C16	0.0708 (12)	0.0523 (11)	0.0663 (12)	−0.0143 (9)	−0.0142 (10)	−0.0139 (9)
C17	0.0611 (11)	0.0810 (14)	0.0456 (10)	0.0059 (10)	−0.0158 (8)	−0.0016 (9)
C18	0.157 (2)	0.0432 (10)	0.0560 (11)	0.0000 (13)	−0.0162 (13)	−0.0037 (9)

### Geometric parameters (Å, º)

**Table:**

S1—C1	1.6634 (16)	C9—C10	1.412 (2)
O1—C2	1.212 (2)	C10—C11	1.366 (2)
O2—C4	1.2145 (19)	C11—H11	0.9300
O3—C7	1.3542 (19)	C12—C13	1.499 (3)
O3—C16	1.424 (2)	C12—H12A	0.9700
O4—C9	1.3478 (19)	C12—H12B	0.9700
O4—C17	1.431 (2)	C13—H13A	0.9600
O5—C10	1.368 (2)	C13—H13B	0.9600
O5—C18	1.408 (2)	C13—H13C	0.9600
N1—C1	1.365 (2)	C14—C15	1.496 (3)
N1—C2	1.407 (2)	C14—H14A	0.9700
N1—C12	1.487 (2)	C14—H14B	0.9700
N2—C1	1.384 (2)	C15—H15A	0.9600
N2—C4	1.407 (2)	C15—H15B	0.9600
N2—C14	1.480 (2)	C15—H15C	0.9600
C2—C3	1.466 (2)	C16—H16A	0.9600
C3—C5	1.377 (2)	C16—H16B	0.9600
C3—C4	1.448 (2)	C16—H16C	0.9600
C5—C6	1.426 (2)	C17—H17A	0.9600
C5—H5	0.9300	C17—H17B	0.9600
C6—C11	1.415 (2)	C17—H17C	0.9600
C6—C7	1.421 (2)	C18—H18A	0.9600
C7—C8	1.389 (2)	C18—H18B	0.9600
C8—C9	1.380 (2)	C18—H18C	0.9600
C8—H8	0.9300		
			
C7—O3—C16	119.70 (14)	N1—C12—C13	111.77 (15)
C9—O4—C17	118.14 (15)	N1—C12—H12A	109.3
C10—O5—C18	116.88 (14)	C13—C12—H12A	109.3
C1—N1—C2	124.63 (13)	N1—C12—H12B	109.3
C1—N1—C12	119.84 (14)	C13—C12—H12B	109.3
C2—N1—C12	115.49 (14)	H12A—C12—H12B	107.9
C1—N2—C4	125.06 (13)	C12—C13—H13A	109.5
C1—N2—C14	119.37 (13)	C12—C13—H13B	109.5
C4—N2—C14	115.52 (13)	H13A—C13—H13B	109.5
N1—C1—N2	116.84 (14)	C12—C13—H13C	109.5
N1—C1—S1	121.91 (12)	H13A—C13—H13C	109.5
N2—C1—S1	121.25 (12)	H13B—C13—H13C	109.5
O1—C2—N1	118.63 (15)	N2—C14—C15	112.36 (15)
O1—C2—C3	123.93 (16)	N2—C14—H14A	109.1
N1—C2—C3	117.44 (14)	C15—C14—H14A	109.1
C5—C3—C4	127.53 (14)	N2—C14—H14B	109.1
C5—C3—C2	113.68 (14)	C15—C14—H14B	109.1
C4—C3—C2	118.78 (14)	H14A—C14—H14B	107.9
O2—C4—N2	117.66 (14)	C14—C15—H15A	109.5
O2—C4—C3	125.48 (15)	C14—C15—H15B	109.5
N2—C4—C3	116.85 (13)	H15A—C15—H15B	109.5
C3—C5—C6	138.77 (14)	C14—C15—H15C	109.5
C3—C5—H5	110.6	H15A—C15—H15C	109.5
C6—C5—H5	110.6	H15B—C15—H15C	109.5
C11—C6—C7	116.80 (14)	O3—C16—H16A	109.5
C11—C6—C5	126.91 (14)	O3—C16—H16B	109.5
C7—C6—C5	116.29 (14)	H16A—C16—H16B	109.5
O3—C7—C8	122.56 (14)	O3—C16—H16C	109.5
O3—C7—C6	116.54 (14)	H16A—C16—H16C	109.5
C8—C7—C6	120.90 (15)	H16B—C16—H16C	109.5
C9—C8—C7	120.58 (14)	O4—C17—H17A	109.5
C9—C8—H8	119.7	O4—C17—H17B	109.5
C7—C8—H8	119.7	H17A—C17—H17B	109.5
O4—C9—C8	124.58 (15)	O4—C17—H17C	109.5
O4—C9—C10	115.65 (15)	H17A—C17—H17C	109.5
C8—C9—C10	119.77 (14)	H17B—C17—H17C	109.5
C11—C10—O5	125.18 (15)	O5—C18—H18A	109.5
C11—C10—C9	119.67 (15)	O5—C18—H18B	109.5
O5—C10—C9	115.14 (14)	H18A—C18—H18B	109.5
C10—C11—C6	122.25 (14)	O5—C18—H18C	109.5
C10—C11—H11	118.9	H18A—C18—H18C	109.5
C6—C11—H11	118.9	H18B—C18—H18C	109.5
			
C2—N1—C1—N2	1.4 (2)	C3—C5—C6—C7	178.82 (18)
C12—N1—C1—N2	179.25 (15)	C16—O3—C7—C8	1.8 (3)
C2—N1—C1—S1	−178.14 (14)	C16—O3—C7—C6	−178.93 (16)
C12—N1—C1—S1	−0.3 (2)	C11—C6—C7—O3	179.37 (14)
C4—N2—C1—N1	−6.7 (2)	C5—C6—C7—O3	−1.0 (2)
C14—N2—C1—N1	175.91 (14)	C11—C6—C7—C8	−1.3 (2)
C4—N2—C1—S1	172.90 (12)	C5—C6—C7—C8	178.27 (14)
C14—N2—C1—S1	−4.5 (2)	O3—C7—C8—C9	179.83 (15)
C1—N1—C2—O1	−178.32 (19)	C6—C7—C8—C9	0.6 (2)
C12—N1—C2—O1	3.8 (3)	C17—O4—C9—C8	7.1 (3)
C1—N1—C2—C3	2.5 (3)	C17—O4—C9—C10	−173.28 (16)
C12—N1—C2—C3	−175.38 (16)	C7—C8—C9—O4	−179.43 (15)
O1—C2—C3—C5	−2.0 (3)	C7—C8—C9—C10	1.0 (2)
N1—C2—C3—C5	177.12 (15)	C18—O5—C10—C11	10.0 (3)
O1—C2—C3—C4	179.2 (2)	C18—O5—C10—C9	−171.0 (2)
N1—C2—C3—C4	−1.7 (3)	O4—C9—C10—C11	178.64 (15)
C1—N2—C4—O2	−172.69 (16)	C8—C9—C10—C11	−1.7 (2)
C14—N2—C4—O2	4.8 (2)	O4—C9—C10—O5	−0.4 (2)
C1—N2—C4—C3	7.3 (2)	C8—C9—C10—O5	179.17 (15)
C14—N2—C4—C3	−175.18 (14)	O5—C10—C11—C6	179.94 (16)
C5—C3—C4—O2	−1.4 (3)	C9—C10—C11—C6	1.0 (2)
C2—C3—C4—O2	177.22 (18)	C7—C6—C11—C10	0.5 (2)
C5—C3—C4—N2	178.57 (15)	C5—C6—C11—C10	−178.98 (16)
C2—C3—C4—N2	−2.8 (2)	C1—N1—C12—C13	−89.7 (2)
C4—C3—C5—C6	−1.4 (3)	C2—N1—C12—C13	88.3 (2)
C2—C3—C5—C6	179.87 (18)	C1—N2—C14—C15	90.12 (19)
C3—C5—C6—C11	−1.6 (3)	C4—N2—C14—C15	−87.55 (18)

### Hydrogen-bond geometry (Å, º)

**Table:**

*D*—H···*A*	*D*—H	H···*A*	*D*···*A*	*D*—H···*A*
C13—H13*C*···O2^i^	0.96	2.58	3.455 (2)	152

Symmetry code: (i) −*x*, *y*+1/2, −*z*+1/2.
